# Deamidated gluten: specific detection and characterisation with INRA-DG1 mAb

**DOI:** 10.1186/2045-7022-5-S3-P136

**Published:** 2015-03-30

**Authors:** Olivier Tranquet, Manon Pietri, Fiona Francini, Maxime Pecquet, Annabelle Blanchet, Valérie Echasserieau-Laporte, Colette Larré, Sandra Denery-Papini

**Affiliations:** 1 Institut National de la Recherche Agronomique, UR1268 BIA, Nantes, France

## 

Diversification of gluten applications was achieved through the production of water-soluble gluten. Deamidation, one of the methods for this purpose, may be obtained with either chemical or enzymatic treatment and lead to the conversion of glutamines into glutamic acids. These types of products can be found both in cosmetics and food.

From the 2000's severe allergic reactions to deamidated gluten (DG) have been reported in individuals although they were tolerant to native wheat proteins. Furthermore, when included in a facial soap sold in Japan, DG has been shown to be involved in a sensitization mechanism enabling both hydrolysed and native proteins to elicit severe skin reactions and food allergy in more than 1300 individuals. Recently, specific epitopes linked to this allergy were identified.[1] Up to now there no satisfying analytical method to detect DG. This study aims to produce new antibodies for characterising and detecting DG in food.

Based on the main epitope involved in allergy to DG, mouse monoclonal antibodies were produced and characterized by ELISA, WB and pepscan and compared with IgE reactivities of patients. Interestingly, all mAbs specifically bind deamidated wheat proteins with high affinity and without any reaction to native proteins. Pepscan analysis on gliadin repeated sequences more or less deamidated showed that only peptides deamidated at least at 50% were recognized. However INRA-DG mAb is able to bind deamidated gliadins at a level as low as 15% suggesting that among the repetitive domain some peptides were highly deamidated.

A competitive ELISA assay based on INRA-DG1 mAb was developed enabling accurate DG detection below the 20 ppm threshold. When compared to the reference ELISA method based on the R5 antibody it appears that INRA-DG1 assay and R5 are complementary. Moreover, no cross reactivity to native flours from wheat, rye, barley, oat, rice, maize and soy were detected.
